# Melatonin enhances osteogenic differentiation of dental pulp mesenchymal stem cells by regulating MAPK pathways and promotes the efficiency of bone regeneration in calvarial bone defects

**DOI:** 10.1186/s13287-022-02744-z

**Published:** 2022-02-19

**Authors:** Ya-Hui Chan, Kuo-Ning Ho, Yu-Chieh Lee, Meng-Jung Chou, Wei-Zhen Lew, Haw-Ming Huang, Pin-Chuang Lai, Sheng-Wei Feng

**Affiliations:** 1 School of Oral Hygiene, College of Oral Medicine, Taipei Medical University, Taipei, Taiwan; 2 School of Dentistry, College of Oral Medicine, Taipei Medical University, No. 250, Wuxing St., Taipei, 11031 Taiwan; 3 Department of Obstetrics and Gynecology, Taipei Medical University Hospital, Taipei, Taiwan; 4 Department of General Dentistry & Oral Medicine, School of Dentistry, University of Louisville, Louisville, USA; 5 Division of Prosthodontics, Department of Dentistry, Taipei Medical University Hospital, Taipei, Taiwan

**Keywords:** Melatonin, Dental pulp mesenchymal stem cells, Osteogenesis, COX2/NF-κB, p38/ERK signaling pathway, Bone regeneration, Calvarial bone defect

## Abstract

**Background:**

Mesenchymal stem cell (MSC)-based tissue engineering plays a major role in regenerative medicine. However, the efficiency of MSC transplantation and survival of engrafted stem cells remain challenging. Melatonin can regulate MSC biology. However, its function in the osteogenic differentiation of dental pulp-derived MSCs (DPSCs) remains unclear. We investigated the effects and mechanisms of melatonin on the osteogenic differentiation and bone regeneration capacities of DPSCs.

**Methods:**

The biological effects and signaling mechanisms of melatonin with different concentrations on DPSCs were evaluated using a proliferation assay, the quantitative alkaline phosphatase (ALP) activity, Alizarin red staining, a real-time polymerase chain reaction, and a western blot in vitro cell culture model. The in vivo bone regeneration capacities were assessed among empty control, MBCP, MBCP + DPSCs, and MBCP + DPSCs + melatonin preconditioning in four-created calvarial bone defects by using micro-computed tomographic, histological, histomorphometric, and immunohistochemical analyses after 4 and 8 weeks of healing.

**Results:**

In vitro experiments revealed that melatonin (1, 10, and 100 μM) significantly and concentration-dependently promoted proliferation, surface marker expression (CD 146), ALP activity and extracellular calcium deposition, and osteogenic gene expression of DPSCs (*p* < 0.05). Melatonin activated the protein expression of ALP, OCN, and RUNX-2 and inhibited COX-2/NF-κB expression. Furthermore, the phosphorylation of mitogen-activated protein kinase (MAPK) p38/ERK signaling was significantly increased in DPSCs treated with 100 μM melatonin, and their inhibitors significantly decreased osteogenic differentiation. In vivo experiments demonstrated that bone defects implanted with MBCP bone-grafting materials and melatonin-preconditioned DPSCs exhibited significantly greater bone volume fraction, trabecular bone structural modeling, new bone formation, and osteogenesis-related protein expression than the other three groups at 4 and 8 weeks postoperatively (*p* < 0.05).

**Conclusions:**

These results suggest that melatonin promotes the proliferation and osteogenic differentiation of DPSCs by regulating COX-2/NF-κB and p38/ERK MAPK signaling pathways. Preconditioning DPSCs with melatonin before transplantation can efficiently enhance MSCs function and regenerative capacities.

## Introduction

Mesenchymal stem cells (MSCs) have self-renewal and multilineage differentiation capacities, including osteoblasts, chondrocytes, and adipocytes [1]. They promote tissue regeneration during bone repair in osteoporosis, bone fracture, osteoarthritis, and bone defects and are considered a good cell source in regenerative medicine [2, 3]. Among various MSC sources, dental pulp-derived MSCs (DPSCs) have been used in tissue engineering and regenerative medicine due to their easy isolation and minimally invasive harvesting from discarded premolars or third molars [4–6]. DPSCs have strong odontogenic, osteogenic, and angiogenic potential [7–9]. Compared with bone marrow-derived MSCs (BMSCs), DPSCs can survive in vitro for a longer time; exhibit a higher growth rate; and produce more extracellular matrix, mineralized tissue, and bone-like trabecular structures with less immunosuppressive properties [7, 10–12]. Animal studies have also confirmed that DPSCs can promote bone regeneration and osseointegration [5, 10, 13].

However, the efficiency of transplanted MSCs is easily affected by the wound condition, bone defect size, and recipient health. Many intrinsic and extrinsic factors can impair the survival rate, regeneration, and differentiation capacities of the transplanted MSCs, including lack of oxygen in the local area, excessive oxidative stress after tissue damage, insufficient blood nutrient supply, excessive cell metabolites, bacterial infection or by-products (such as lipopolysaccharide), and autoimmune inflammation response [14, 15]. MSC-based tissue regeneration can become considerably more effective if the transplanted MSCs can maintain survival or activity until angiogenesis around the bone defect occurs to provide sufficient oxygen and nutrients and remove metabolites [15, 16]. Moreover, disease, age, and long-term expansion of MSCs influence their proliferation and differentiation characteristics [17, 18]. Therefore, MSC preconditioning may be a strategy for increasing the effectiveness of MSC transplantation [17, 19, 20].

MSCs can be treated with cytokines, growth factors, hypoxic conditions, mechanical stimulation, and small molecules to promote their immunomodulatory, regenerative, angiogenic, and antiapoptotic capacities [21–24]. Among these factors, melatonin (*N*-acetyl 5-methoxytriptamine [MLT]) can serve as a critical role in MSC-based regenerative therapy [25]. Melatonin regulates pathophysiological processes, including the circadian cycle, cardiovascular function, visceral function, and metabolic processes related to healing, and inhibits tumor growth [26–29]. It also has immunomodulatory, anti-inflammatory, antioxidant, and free-radical scavenging abilities [30]. MSCs pretreated with melatonin have antioxidant, antiapoptotic, antiaging, and cytoprotective effects and stemness maintenance properties [17].

Melatonin not only promotes osteoblast or MSCs differentiation by regulating osteogenic gene expression, but also regulates MSCs-mediated osteoclastogenesis by indirectly secreting cytokines [31, 32]. In vitro studies have confirmed that melatonin can activate the expression of *Runx2, OCN,* and *BMP* genes of MSCs and promote the osteogenic differentiation and mineralization abilities of osteoblasts via the BMP, ERK, Wnt, and PKA/PKC signaling pathways [33, 34]. A mouse study demonstrated that daily intraperitoneal melatonin injections can promote the growth of cortical bone [35]. In addition, the administration of 5% melatonin in the alveolar bone socket can promote bone regeneration and osseointegration around the dental implant [36, 37].

Although melatonin exerts biological effects on osteoblasts and MSCs, the effects of melatonin on the proliferation, osteogenic differentiation, mineralization, osteogenic gene expression, and signaling mechanisms of DPSCs remain unclear [38, 39]. Moreover, the bone regeneration capacities of melatonin-preconditioned DPSCs combined with bone-grafting materials in calvarial bone defects need to be clarified. A detailed understanding of melatonin-treated DPSCs both in vitro and in vivo will be beneficial for future MSC therapy and maxillofacial bone reconstruction in clinics. Therefore, the present study (1) evaluated the effects of different concentrations of melatonin on the proliferation, osteogenic differentiation, and mineralization of DPSCs; (2) explored the underlying molecular mechanisms triggered by melatonin on DPSCs during osteogenic differentiation; and (3) evaluated the bone regeneration capacities and osteogenesis-related protein expression of preconditioning DPSCs with melatonin in calvarial bone defects.

## Materials and methods

### DPSCs culture and chemical reagents

All experimental protocols for in vitro cell isolation and in vivo study were performed under ethical approval from the Institutional Animal Care and Use Committee of Taipei Medical University, Taipei, Taiwan (approval no. LAC-2017-0514). DPSCs were isolated from freshly extracted teeth, as described previously [5, 8, 40]. In brief, incisors were extracted from different rabbits, and the dental pulp tissues were carefully harvested. After the dental pulp tissues were washed with phosphate-buffered saline (PBS) three times and minced into pieces, smaller pieces of pulp tissue were cultured in 3.5-cm-diameter Petri dishes with growth medium (GM) containing α-minimum essential medium (α-MEM; Thermo Fisher Scientific, Waltham, MA, USA) supplemented with 15% fetal bovine serum, with 1000 U/mL penicillin, 1000 μg/mL streptomycin, and 0.25 μg/mL amphotericin B (Thermo Fisher Scientific, Waltham, MA, USA) in an incubator at 37 °C with 5% CO_2_. The culture medium was refreshed every 3 days. At approximately 70% confluence, the outgrowth cells were detached using 0.25% trypsin/EDTA. The cells were passed through a 70-μm mesh nylon filter (BD Biosciences, Bedford, MA, USA) to obtain a single-cell suspension. DPSCs between passages 3 and 6 were used in the following experiments. In addition, the multilineage differentiation capacities of DPSCs were confirmed using osteogenic, chondrogenic, and adipogenic induction media.

Melatonin (M5250), SB203580 (p38 mitogen-activated protein kinase [MAPK] pathway inhibitor), and PD98059 (ERK/MAPK pathway inhibitor) were purchased from Sigma-Aldrich (St Louis, MO, USA) and dissolved in dimethylsulfoxide (DMSO) and then diluted in PBS immediately before use. Melatonin (0, 1, 10, and 100 μM) was directly added to the GM and osteogenic differentiation medium (OM) to determine its effects on DPSCs.

### MTT assay

DPSCs were seeded in 48-well plates (2 × 10^4^ cells/well) and grown for 24 h in GM under standard conditions. Afterward, 100, 10, and 1 μM melatonin was applied. Following melatonin treatment for 1, 3, and 5 days, MTT [3-(4,5-dimenthylthiazol-2-yl)-2,5-diphenyltetrasoliumbromide] (Roche Applied Science, Mannheim, Germany) solution (5 mg/mL) was added. After 4 h of culture, the optical density of formazan crystal dissolved with DMSO was detected at a wavelength of 570 nm with a reference wavelength of 690 nm by a microplate reader (EZ read 2000, Biochrom, Cambridge, UK). The optical density values of each well represent the viability of DPSCs.

### Surface marker expression

To characterize the MSCs obtained and the effects of melatonin, the surface molecule markers of DPSCs were evaluated using flow cytometry. DPSCs were seeded in 10-mm dishes and exposed to 0, 100, 10 and 1 μM melatonin for 24 h. The DPSCs from each group were detached with trypsin–EDTA and centrifuged at 1000 rpm for 5 min. The collected DPSCs were then incubated with fluorescein isothiocyanate (FITC)-conjugated antibodies against antigens CD90, CD105, CD44, CD146, CD34, and CD45 for 30 min in the dark at 4 °C. The stained DPSCs were washed twice with PBS and fixed in 4% paraformaldehyde for 10 min at 4 °C. Flow cytometry was performed using a Guava easyCyte flow cytometer (Merck Millipore, Billerica, MA, USA), while the data were analyzed using FlowJo software (TreeStar, Ashland, OR, USA). Negative control staining was performed using the FITC-conjugated mouse IgG1 isotype.

### Alkaline phosphatase activity assay

DPSCs were seeded in a 24-well plate at 2.0 × 10^4^ cells/mL in α-MEM [41]. After 24 h of incubation, the α-MEM was replaced with GM and OM containing different concentrations of melatonin (0, 100, 10, and 1 μM) for 1 and 3 days. After further incubation, the DPSCs of each experimental group were washed three times with PBS, and then, 300 μL CelLytic P Cell Lysis Reagent (Sigma-Aldrich) was added to each sample to release the alkaline phosphatase (ALP) from the cell.

Thereafter, 50 μL of each cell lysate was mixed with 250 μL of *p*-nitrophenyl phosphate (pNPP) in the 96-well plate for 30 min at 37 °C. ALP activity was measured by detecting optical density values at 405 nm. ALP activity was normalized to the cellular protein, which was determined with the Bio-Rad protein assay kit (Bio-Rad, USA). Finally, the ALP enzymatic activity was calculated using the two measured parameters, and the results are expressed as mM p-nitrophenol produced per minute per microgram of protein.

### Alizarin red staining and accumulated calcium assay

For osteoblastic differentiation, when reaching 80% confluence, the DPSCs were cultured in OM containing 15% fetal bovine serum, 1000 U/mL penicillin, 1000 μg/mL streptomycin, 0.25 μg/mL amphotericin B, 0.01 μM dexamethasone, and 1.8 mM KH_2_PO_4_. The DPSCs were cultured in GM and OM containing different concentrations of melatonin (0, 100, 10, and 1 μM) for 7 and 14 days. The culture medium was changed once every 3 days. After 7 and 14 days of induction, DPSCs were gently washed three times with PBS solution, fixed with 4% paraformaldehyde for 10 min and stained with 2% Alizarin red S (Sigma-Aldrich) for another 15 min at 37 °C. Alizarin red S stained calcium-rich deposits and matrix mineralization deposition secreted by DPSCs. The stained cells and matrix mineralization deposition were observed under an optical microscope (Eclipse TS100; Nikon Corporation, Tokyo, Japan) and captured by a CMOS camera with SPOT Advance imaging software (SPOT Idea, Diagnostic Instruments, Sterling Heights, MI, USA). For the quantitative assay, the stained cells and matrix were dissolved using 10% acetic acid and transferred to Eppendorf tubes. After heating to 85 °C in a digital dry bath incubator for 10 min and then cooling for 5 min in an ice bath, the dissolved nodules were centrifuged at 15,000 × g for 15 min. Finally, 10% ammonium hydroxide was added to neutralize the acetic acid solution. The mixture was then analyzed spectrophotometrically at 405 nm.

To confirm the effect of melatonin and investigate the involvement of the MAPK pathway, the cells were pretreated with a specific p38 inhibitor (SB203580; Sigma-Aldrich) and ERK inhibitor (PD98059; Sigma-Aldrich) for 2 h and then co-cultured with melatonin. Next, osteogenic differentiation assay and RT-PCR were conducted.

### Reverse transcription quantitative PCR

Total RNA was isolated using a Total RNA Mini Kit (NovelGene Biotech, Taipei, Taiwan) after 72 h of cell culture with GM and OM for cDNA synthesis and qPCR analysis of the expression of osteogenesis-related genes, according to the manufacturer’s instructions. For mRNA detection, cDNA was generated using a high-capacity cDNA Reverse Transcription Kit (Applied Biosystems, Foster City, CA, USA). The cDNA was amplified using a real-time DNA thermal analyzer (TurboCycler Lite, Blue-Ray Biotech, Taipei, Taiwan). The quantitative real-time PCRs were performed in 20-μL reactions using a FastStart Universal SYBR Green Master kit (Roche Applied Science) in 48-well plates sealed with adhesive films in the Eco Real-Time PCR System (Illumina, San Diego CA, USA). The specific primer sequences of the osteogenic genes are presented in Table 1. The comparative ΔCt method was used to calculate the relative expression of ALP, bone morphogenetic protein-2 (BMP-2), osteocalcin (OCN), and runt-related transcription factor 2 (RUNX2) genes. The housekeeping gene glyceraldehyde 3-phosphate dehydrogenase (GAPDH) was used for normalization. The relative expression levels of the genes were normalized and analyzed using the 2 − ΔΔCT method [5].

**Table 1:** Primers used for RT-PCR

Gene	Type	Primers	Accession	Product length
ALP	Forward	5′-ACTGTGGACTACCTCTTG-3′	XM_017346489	76
	Reverse	5′-GGTCAGTGATGTTGTTCC-3′		
BMP-2	Forward	5′-CGTGAGGATTAGCAGGTCTTTG-3′	NM_001082650	127
	Reverse	5′-TTTCGCTTGACGCTTTTCTC-3′		
Osteocalcin	Forward	5′- ACTCTTGTCGCCCTGCTG-3′	XM_002715383	116
	Reverse	5′-CTGCCCTCCCTCTTGGAC-3′		
Runx2	Forward	5′-TCAGGCATGTCCCTCGGTAT-3′	XM_017345160	54
	Reverse	5′-TGGCAGGTAGGTATGGTAGTGG-3′		
GAPDH	Forward	5′-GCCTGGAGAAAGCTGCTAAGT-3′	NM_001082253	133
	Reverse	5′-GAGTGGGTGGCACTGTTGAA-3′		

### Protein isolation and Western blotting

For Western blotting, cell lysates were isolated from 80% confluent culture plates by direct lysis of DPSCs at 4 °C with radioimmunoprecipitation assay (RIPA) buffer supplemented with protease and phosphatase inhibitors (Sigma-Aldrich). The total protein concentration was quantified using the Pierce BCA protein assay kit (Thermo Fisher Scientific). The extracted cell lysates (10 μg of total proteins) were resolved with sodium dodecyl sulfate polyacrylamide gel electrophoresis (SDS-PAGE) and transferred to a polyvinylidene difluoride membrane (Millipore, Bedford, MA, USA). After incubation in 5% bovine serum albumin (BSA) at room temperature, the membranes were incubated overnight with primary antibodies at 4 °C. The alkaline phosphatase/ALPP (#8B6), BMP-2 (#9E10G12), osteocalcin (#190125), and RUNX2/CBFA1 (#CL0232) antibodies were from Novus Biologicals (OCN Littleton, CO, USA). The COX2 (#37843) and NF-κB (#6956), p38 (#9212), p-p38 (#9216), JNK (#9252), p-JNK (#9255), ERK (#9102), and p-ERK (#4696) antibodies were from Cell Signaling Technology (Beverly, MA, USA). All antibodies were diluted 1:1000. Horseradish peroxidase (HRP)-conjugated immunoglobin G (IgG) was used as the secondary antibody (1:2000, Cell Signaling Technology). The immunostained protein bands were visualized using an ECL-Plus detection kit (PerkinElmer Life Sciences, Boston, MA, USA). The proteins were normalized to β-actin, and the intensity of each band was quantified using ImageJ software (National Institutes of Health, Bethesda, MD, USA).

### Animals and ethics

Adult male New Zealand white rabbits weighing between 3.5 and 4.0 kg were used in this study and subject to a calvarial defect assay. All experimental procedures were conducted in compliance with ethics approval from the Institutional Animal Care and Use Committee of Taipei Medical University, Taipei, Taiwan (approval no. LAC-2017-0514) under the ARRIVE guidelines [42, 43]. Animals were individually housed in the Central Animal Facility at Taipei Medical University under an adjusted climate (temperature 22 ± 2 °C, humidity 30–60% ± 5%, a 12/12 h light/dark cycle) and free access to a standard diet and drinking water.

### Experimental materials and surgical procedures

The animals were anesthetized using an intramuscular injection of tiletamine-zolazepam at a dose of 15 mg/kg (Zoletil 50, Virbac, Carros Cedex, France). The dorsal area of the rabbit cranium was shaved before surgery. After the surgical site was disinfected with beta-iodine, local anesthesia with 1.8 mL of 2% lidocaine (1: 100,000 epinephrine) was provided. The calvarial bone was exposed after midline skin incision, muscle dissection, and periosteal elevation. Four separated circular bone defects were created using an outer diameter 6-mm trephine bure under copious irrigation with sterile saline (Fig. 9a). Care was taken to avoid injury to the dura. The following four treatment modalities were randomly allocated to bone defects (*n* = 6 per group): (1) empty control (2) HA/TCP ceramic powder (MBCP, Biomatlante, Vigneux, France), (3) HA/TCP ceramic powder and DPSCs preincubated with OM for 5 days, and (4) HA/TCP ceramic powder and DPSCs preconditioned with OM and 100 μM melatonin for 5 days. DPSCs were seeded homogeneously on HA/TCP ceramic powders and incubated for additional 4 h to allow cell attachment before in vivo implantation. After the operation, the muscle layer was closed with a bioresorbable suture (Vicryl 4.0, Ethicon, Somerville, NJ, USA), and the skin layer was sutured with a nylon suture. Antibiotics (Baytril, Bayer, Leverkusen, Germany) (5.0 mg/kg, subcutaneously, twice a day) and analgesics (Rimadyl, Pfizer, New York, USA) (4.0 mg/kg, subcutaneously, twice a day) were administered postoperatively for 3 days to prevent infection and control pain.

### Micro-computed tomography measurements

The animals were sacrificed after 4 and 8 weeks of healing, and the defects, along with an additional 1 mm of the surrounding tissue, were harvested from the host bone. After the harvested samples were fixed in 10% neutral buffered formalin for 3–5 days, they were processed and scanned with micro-computed tomography (CT) (Bruker Skyscan 1172, Bruker, Kontich, Belgium), as described previously (Lee et al. 2019). In brief, the samples were scanned using micro-CT with the X-ray source present at a voltage of 50 kV, an electric current of 100 mA, and a pixel resolution of 18 μm with a 0.5-mm aluminum filter. Next, three-dimensional (3D) image models were adjusted and reconstructed using DataViewer software (Bruker). After determining the volume of interest and optimized threshold, parameters including bone volume density (bone volume/total tissue volume, BV/TV%), trabecular thickness (Tb.Th mm), and trabecular number (Tb.N mm^−1^) were measured using the CTAn analysis program (Bruker Skyscan 1172).

### Histology and histomorphometric analysis

After micro-CT analysis, samples were decalcified in Plank-Rychlo's solution (MUTO Pure Chemicals, Tokyo, Japan) for 7 days, dehydrated in graded ethanol baths (80–100%), and embedded in paraffin. Using a rotary microtome, 4-μm-thick sections were cut from the samples and stained with hematoxylin–eosin (HE; Sigma-Aldrich) and Masson’s trichrome. Histologic observations and images were acquired using a TissueFAXS plus system (TissueGnostics GmbH, Vienna, Austria). For histomorphometric analysis, four sites were randomly selected for each slide, and the new bone formation area of each region was calculated using Image-Pro Plus 6.0 software (Media Cybernetics, Silver Spring, MI, USA).

### Immunohistochemical analysis

The specimens were permeabilized with 0.1% Triton X-100 for 10 min and blocked in 3% BSA for 30 min at room temperature. The tissue specimens were then incubated in a 1:100 diluted anti-osteocalcin solution and a 1:100-diluted anti-runx2 antibody solution (Novus Biologicals, Littleton, CO, USA) at 37 °C. Finally, 3,3′-diaminobenzidine tetrahydrochloride (DAB) (DAKO, Glostrup, Denmark) was used as a substrate, and the specimens were counterstained with hematoxylin. Histologic observations and images were acquired using a TissueFAXS plus system (TissueGnostics GmbH).

### Statistical analysis

All quantitative data are presented as the mean ± standard deviation (SD) from the three experiments. Statistical analysis was performed using a one-way analysis of variance, followed by Tukey’s honest significant difference test. All statistical analyses were conducted using SPSS for Windows (version 19, SPSS, Chicago, IL, USA). *p* < 0.05 (*) and *p* < 0.01 (**) were considered significant.

## Results

### Characterizations of DPSCs

**Fig. 1:**
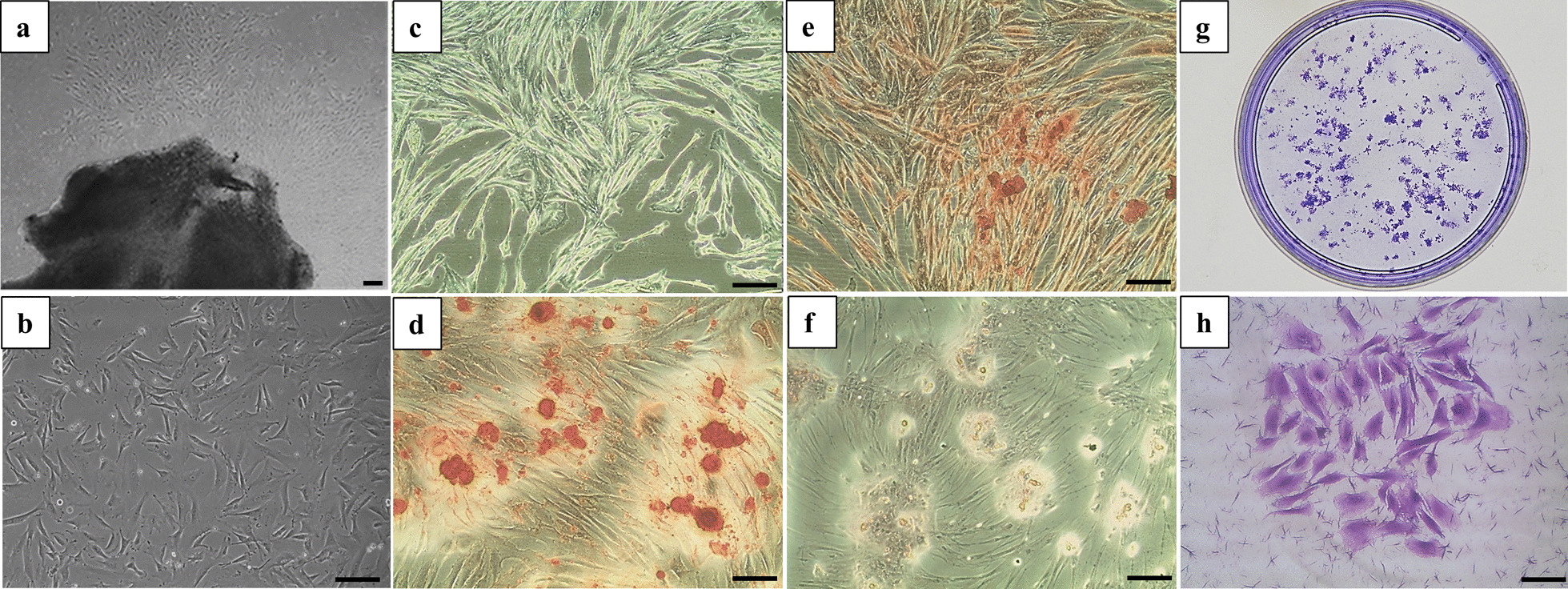
Characterizations and tri-lineage differentiation of DPSCs. **a** DPSCs had a typical fibroblast-like morphology at primary culture and **b**, **c** passage 3. **d** Osteogenic differentiation was confirmed with positive Alizarin red S staining. **e** Chondrogenic differentiation was verified with Safranin O staining. **f** Adipogenic differentiation was confirmed with positive Oil red O staining. **g**, **h** Representative images of colony-forming units for DPSCs. Scale bar = 100 μm

In the in vitro experiments, DPSCs were morphologically homogeneous and fibroblast-like (Fig. 1a, b). Trilineage differentiation of DPSCs was confirmed with osteogenic, chondrogenic, and adipogenic differentiation (Fig. 1c–f). Alizarin red S staining displayed extracellular calcium deposition in the cell layer after 14 days of osteogenic differentiation induction (Fig. 1d). Safranin O staining exhibited positive potential for the chondrogenic phenotype after 14 days of chondrogenic differentiation induction (Fig. 1e). Oil red O staining revealed intracellular lipid accumulation after 21 days of adipogenic differentiation induction (Fig. 1f). Moreover, DPSCs also exhibited the capacity for colony-forming unit formation (Fig. 1g, h).

### Effects of melatonin on DPSC proliferation

**Fig. 2:**
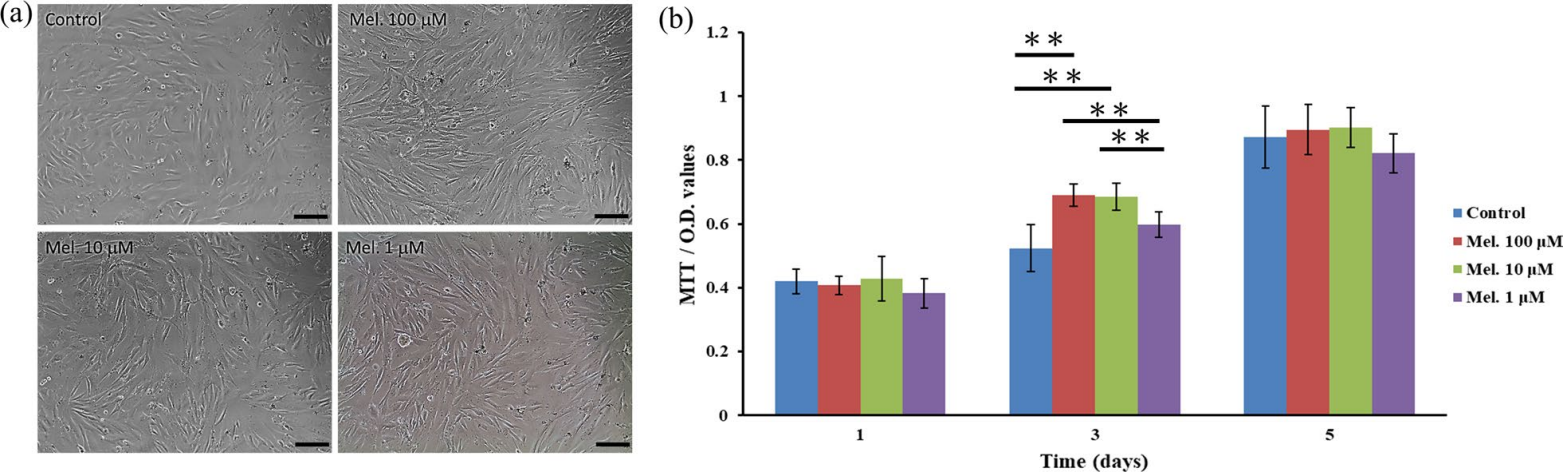
Effect of melatonin on DPSCs proliferation at various concentrations (0, 100, 10, and 1 μM). **a** Melatonin-treated and untreated DPSCs exhibited similar spindle-shaped morphology after 3 days of culture. **b** DPSC proliferation was promoted in the presence of melatonin at 100 and 10 μM compared with the untreated control group. Scale bar = 100 μm. (*p < 0.05, **p < 0.01)

To investigate the effect of melatonin on DPSC proliferation, DPSCs were treated with various concentrations (0, 100, 10, and 1 μM) of melatonin and examined using the MTT assay after 1, 3, and 5 days. MTT assay is used to measure cellular metabolic activity as an indicator of cell viability and proliferation. This colorimetric assay is based on the reduction of a yellow tetrazolium salt (3-(4,5-dimethylthiazol-2-yl)-2,5-diphenyltetrazolium bromide, MTT) to purple formazan crystals by metabolically active cells. The darker the solution dissolved from purple formazan crystals indicated the higher the proliferation of DPSCs. After 3 days of culture, DPSCs displayed a similar spindle shape in all groups (Fig. 2a). Melatonin treatment at 100 and 10 μM can significantly increase proliferation of DPCSs compared with the untreated control cells and 1 μM-treated cells after 3 days of culture (*p* < 0.01) (Fig. 2b).

### Fluorescence-activated cell sorting analysis of melatonin-treated and untreated DPSCs

**Fig. 3:**
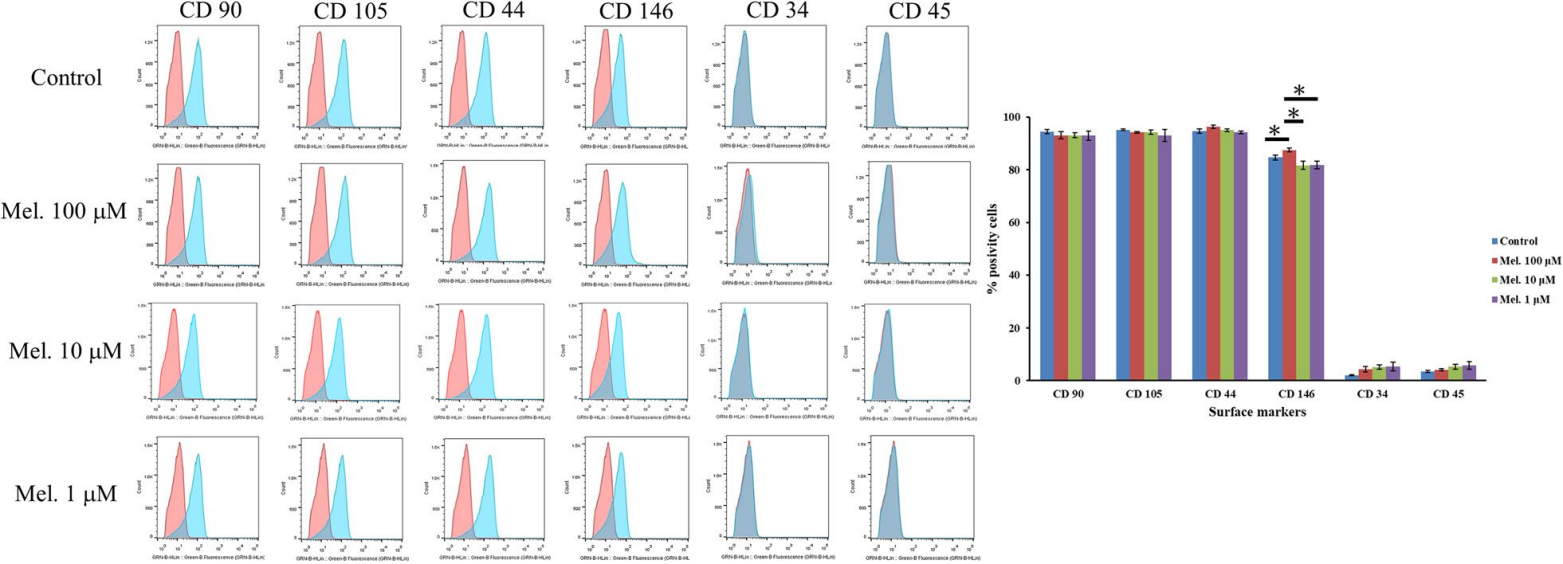
Characterization of surface marker profile for the melatonin-treated (100, 10, and 1 μM) and untreated DPSCs after 24 h of culture. Representative histograms show the percentages of positive cells (blue peaks) versus isotype controls (red peaks). (*p < 0.05, **p < 0.01)

To examine whether melatonin treatment affects the cell surface marker profile of DPSCs, cells were treated with various concentrations (0, 100, 10, and 1 μM) of melatonin for 24 h. As illustrated in Fig. 3, the melatonin-untreated DPSCs positively expressed MSC markers, such as CD90, CD105, CD44, and CD146, while they negatively expressed hematopoietic cell markers, such as CD34 and CD45. Similar to melatonin-untreated DPSCs, DPSCs treated with melatonin exhibited a comparable cell surface expression pattern. In addition, the DPSCs treated with 100 μM melatonin exhibited significantly higher expression of CD146 (87.5% ± 0.7%) than the control (84.6% ± 0.9%), 10 μM melatonin (81.7% ± 1.6%), and 1 μM melatonin groups (81.8% ± 1.4%).

### Melatonin enhanced osteogenic differentiation of DPSCs

**Fig. 4:**
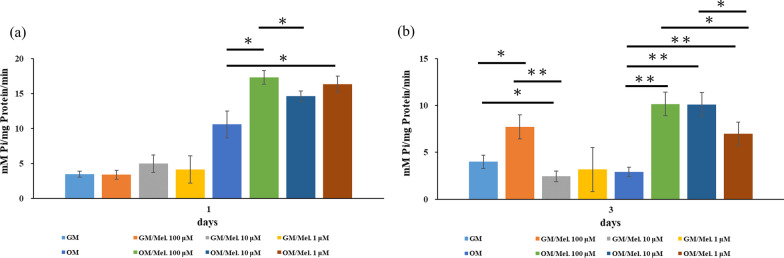
Effect of melatonin (0, 100, 10, and 1 μM) on ALP activity of DPSCs at 1 and 3 days after incubation with and without osteogenic differentiation medium. **a** Melatonin at 100 μM significantly enhanced ALP activity of DPSCs with osteogenic differentiation medium after 1 day of culture. **b** Melatonin at 100, 10, and 1 μM significantly enhanced ALP activity of DPSCs with osteogenic differentiation medium after 3 days of culture. (*ALP* alkaline phosphatase, *GM* growth medium, *OM* osteogenic medium, *Mel* melatonin) (*p < 0.05, **p < 0.01)

The ALP activity of the melatonin-treated DPSCs (100, 10, and 1 μM) was significantly higher than that of the melatonin-untreated DPSCs with OM induction after 1 day of culture (*p* < 0.05) (Fig. 4a). After 3 days of culture, the melatonin-treated DPSCs (100 μM) with GM and OM revealed significantly more ALP activity than the other groups (*p* < 0.05). However, no significant differences in the ALP activity were observed among melatonin-untreated DPSCs and melatonin-treated DPSCs (10 and 1 μM) without osteogenic induction after 1 and 3 days of culture.

**Fig. 5:**
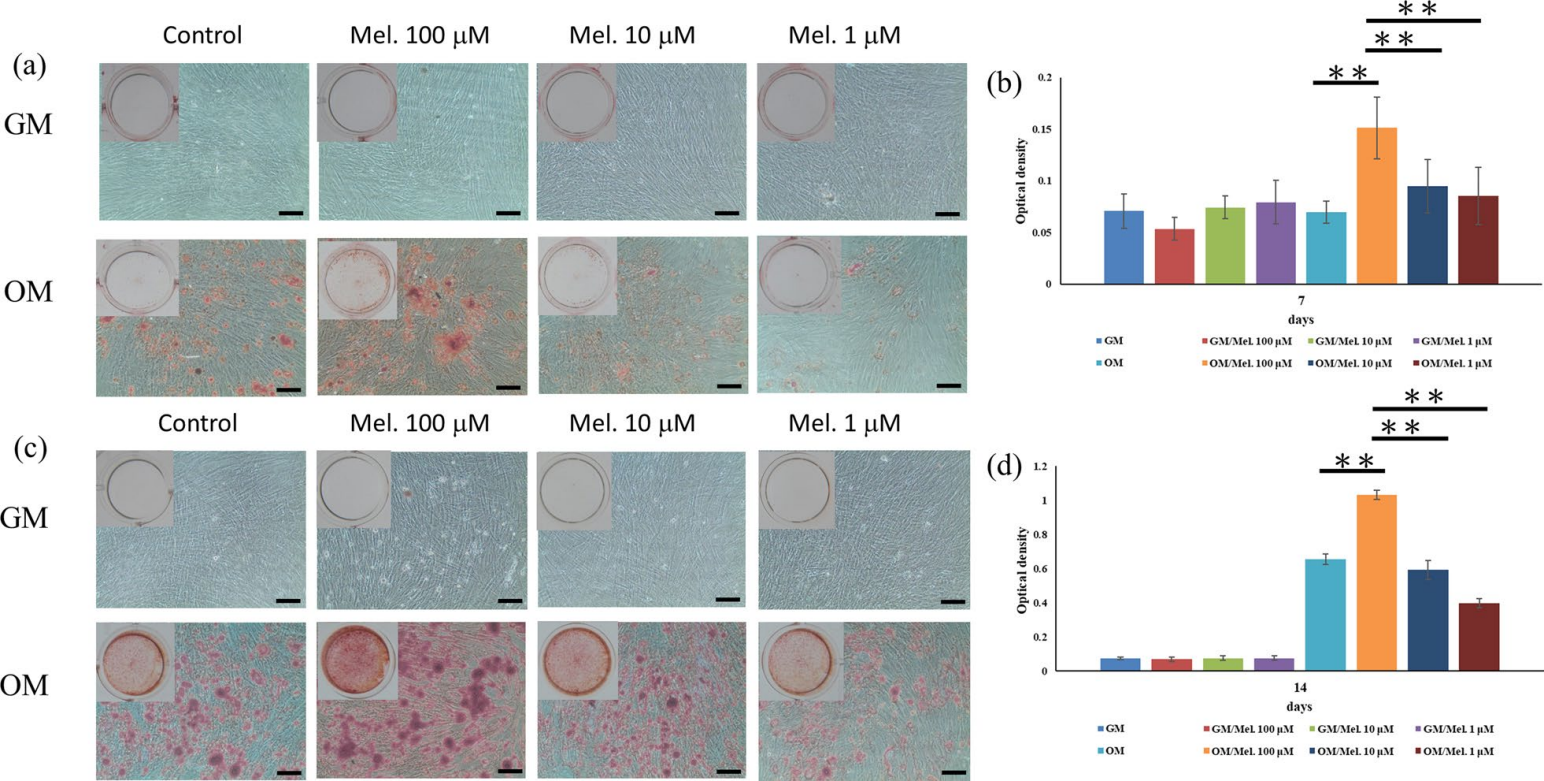
Effects of melatonin (0, 100, 10, and 1 μM) on mineralization of DPSCs at 7 and 14 days after incubation with and without osteogenic differentiation medium. **a**, **c** Extracellular calcium deposition was determined with Alizarin red S staining. **b**, **d** Extracellular calcium deposition was quantified colorimetrically. Melatonin at 100 μM significantly enhanced mineralization of DPSCs with osteogenic differentiation medium. Scale bar = 100 μm. (*GM* growth medium, *OM* osteogenic medium, *Mel* melatonin) (*p < 0.05, **p < 0.01)

Alizarin red S staining was used to quantify the mineral matrix depositions of the melatonin-untreated and treated DPSCs after 7 and 14 days of osteogenic induction culture. As illustrated in Fig. 5a, b, significant differences in the mineral matrix depositions were found between the control group and melatonin-treated groups in the OM experiments. After 7 and 14 days of OM induction, 100 μM melatonin can induce more mineral matrix depositions, while 10 and 1 μM melatonin reveal fewer beneficial effects on the enhancement of osteogenic differentiation for DPSCs. However, in the GM experiments, no significant differences were observed among the experimental groups. This result is consistent with the quantitative Alizarin red S staining data (Fig. 5b), which also shows that osteogenic differentiation of DPSCs was significantly enhanced after treatment with 100 μM melatonin compared with the control group, 10, and 1 μM melatonin-treated groups after 7 and 14 days (*p* < 0.01).

### Melatonin increased the osteogenic gene expression of DPSCs

**Fig. 6:**
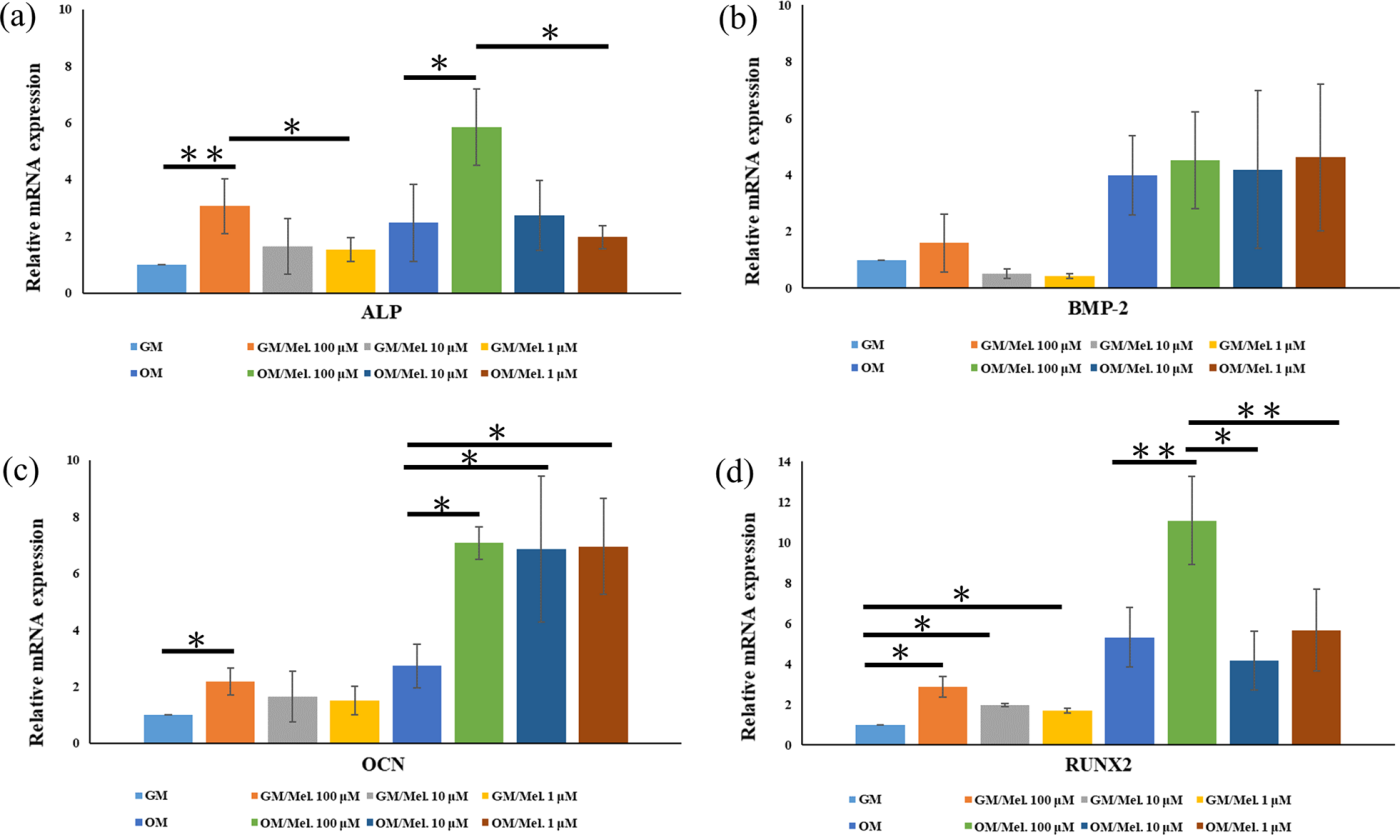
Effects of melatonin (0, 100, 10, and 1 μM) on ALP, BMP-2, OCN, and RUNX2 gene expression in DPSCs at 3 days after incubation with and without osteogenic differentiation medium. Gene expression was measured using real-time PCR. Gene expression was normalized to GAPDH. **a** ALP, **b** BMP-2, **c** OCN, **d** RUNX2. (*GM* growth medium, *OM* osteogenic medium, *Mel* melatonin) (*p < 0.05, **p < 0.01)

The expression of osteogenesis-related genes among melatonin-untreated DPSCs and melatonin-treated DPSCs (100, 10, and 1 μM) with GM and OM induction was analyzed after 3 days of culture. As illustrated in Fig. 6, the expression of osteogenesis-related genes, including ALP, OCN, and Runx-2, was significantly elevated in 100 μM melatonin-treated DPSCs with GM and OM induction compared with melatonin-untreated DPSCs. For BMP-2 gene expression, no significant effect was found among melatonin-untreated DPSCs and melatonin-treated DPSCs (100, 10, and 1 μM) with GM and OM induction. Moreover, 10 and 1 μM melatonin-treated DPSCs with OM induction exhibited significantly higher OCN expression than melatonin-untreated DPSCs (*p* < 0.05).

### Melatonin activated p38/ERK and inhibited NF-κB/COX-2 signaling pathways in DPSCs

**Fig. 7:**
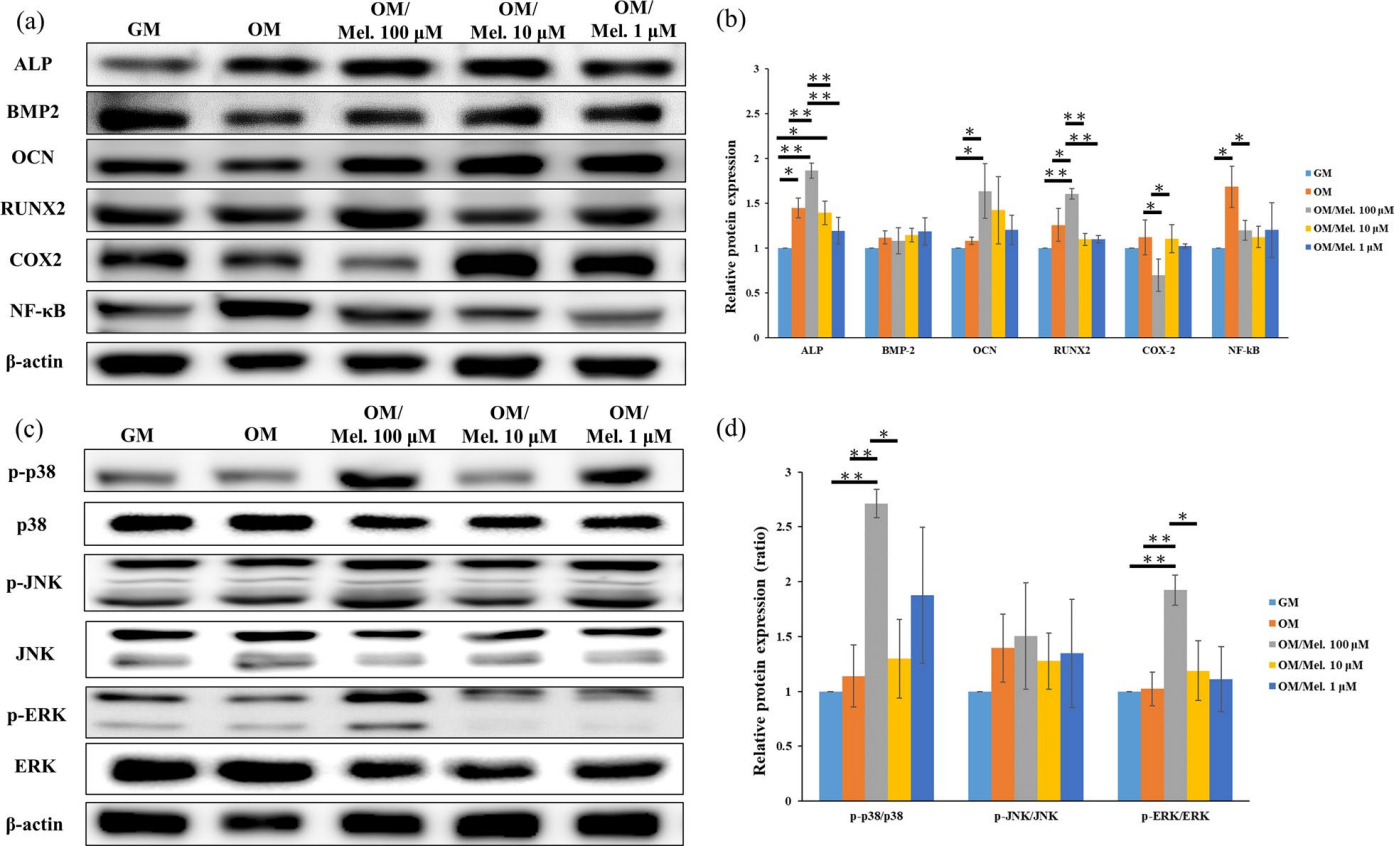
Effects of melatonin (0, 100, 10 and 1 μM) on ALP, BMP-2, OCN, RUNX2, COX-2, NF-κB and MAPK pathway protein level expression in melatonin-untreated DPSCs and melatonin-treated DPSCs with osteogenic differentiation after 24 h incubation. **a**, **b** DPSCs with 100 μM melatonin treatment enhanced ALP, OCN and RUNX2 protein expression and downregulated COX-2 and NF-κB protein expression. **c** DPSCs with 100 μM melatonin treatment induced phosphorylation of p38 and ERK. The phosphorylation of JNK was not observed. **d** Quantitative analysis for the ratios of p-p38/p38, p-JNK/JNK and p-ERK/ERK. (*GM* growth medium, *OM* osteogenic medium, *Mel* melatonin) (*p < 0.05, **p < 0.01)

Western blotting and quantitative analysis of the protein levels of ALP, BMP-2, OCN, RUNX2, COX-2, NF-κB, and MAPK pathways (p-p38, p38, p-JNK, JNK, p-ERK, and ERK) among melatonin-untreated DPSCs and melatonin-treated DPSCs (100, 10, and 1 μM) with OM induction were analyzed after 24 h of culture, and the results are presented in Fig. 7. Western blot analysis revealed that ALP, OCN, and RUNX-2 expression was significantly induced in 100 μM melatonin-treated DPSCs compared with melatonin-untreated DPSCs with OM induction (Fig. 7a, b). In addition, the protein levels of COX-2 and NF-κB were upregulated in DPSCs after osteogenic induction compared with the DPSCs culture in GM. However, this activation was significantly attenuated in the melatonin-treated DPSCs (100, 10, and 1 μM) with osteogenic induction, and 100 μM melatonin-treated DPSCs had the most obvious effect. Furthermore, to explore the role of MAPK pathways during melatonin-mediated enhancement of osteogenic differentiation in DPSCs, the protein levels of p-p38, p38, p-JNK, JNK, p-ERK, and ERK were assayed. No significant differences were observed in the ratio of p-JNK/JNK among the experimental groups (Fig. 7c, d). Notably, the significant expression of p-p38 and p-ERK was activated in 100 μM melatonin-treated DPSCs with OM induction. The ratios of p-p38/p38 and p-ERK/ERK further demonstrated the p-p38 and p-ERK were upregulated by melatonin treatment (Fig. 7c, d).

### Inhibition of p38/ERK MAPK pathways downregulated osteogenic differentiation of melatonin-treated DPSCs with osteogenic induction

**Fig. 8:**
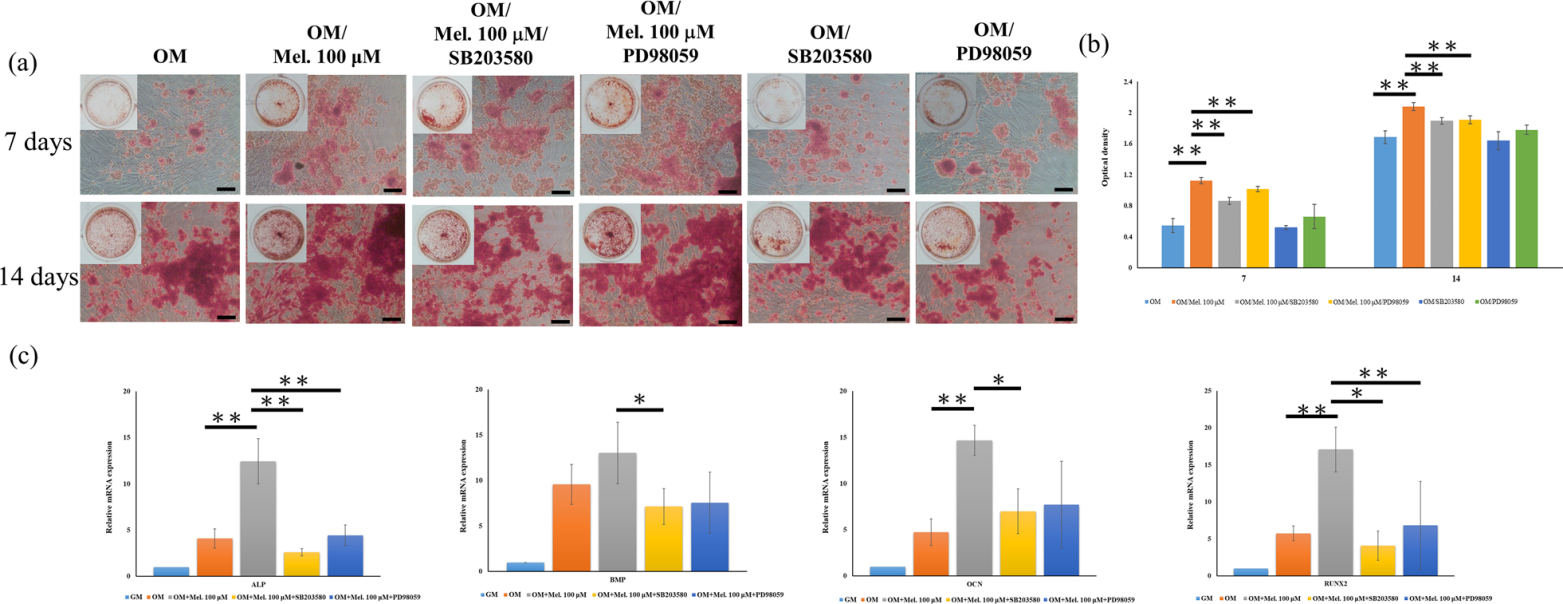
Effects of MAPK pathway inhibitors on osteogenic differentiation of DPSCs treated with 100 μM melatonin at 7 and 14 days after incubation with osteogenic differentiation medium. **a**, **b** SB203580 (p38 MAPK inhibitor) and PD98059 (ERK MAPK inhibitor) significantly reduced the mineral matrix depositions of 100 μM melatonin-treated DPSCs. **c** The gene expression of ALP, BMP-2, OCN, and RUNX2 in 100 μM melatonin-treated DPSCs was suppressed in the experimental groups with SB203580 and PD98059 inhibitors. Scale bar = 100 μm. (*p < 0.05, **p < 0.01)

SB203580 (p38 inhibitor) and PD98059 (ERK inhibitor) were applied for verifying the involvement of MAPK in the melatonin-mediated osteogenic differentiation of DPSCs. DPSCs were firstly treated with SB203580 and PD98059 for 2 h before melatonin treatment. Alizarin red S staining revealed that mineral matrix depositions remarkably decreased in 100 μM melatonin-treated DPSCs with SB203580 and PD98059 inhibitors after 7 and 14 days of osteogenic induction (Fig. 8a, b). After 7 days of osteogenic induction, the gene expression of ALP, BMP-2, OCN, and Runx2 was significantly declined in 100 μM melatonin-treated DPSCs with SB203580 and PD98059 inhibitors (Fig. 8c).

### DPSCs preconditioned with melatonin promoted bone regeneration in vivo

**Fig. 9:**
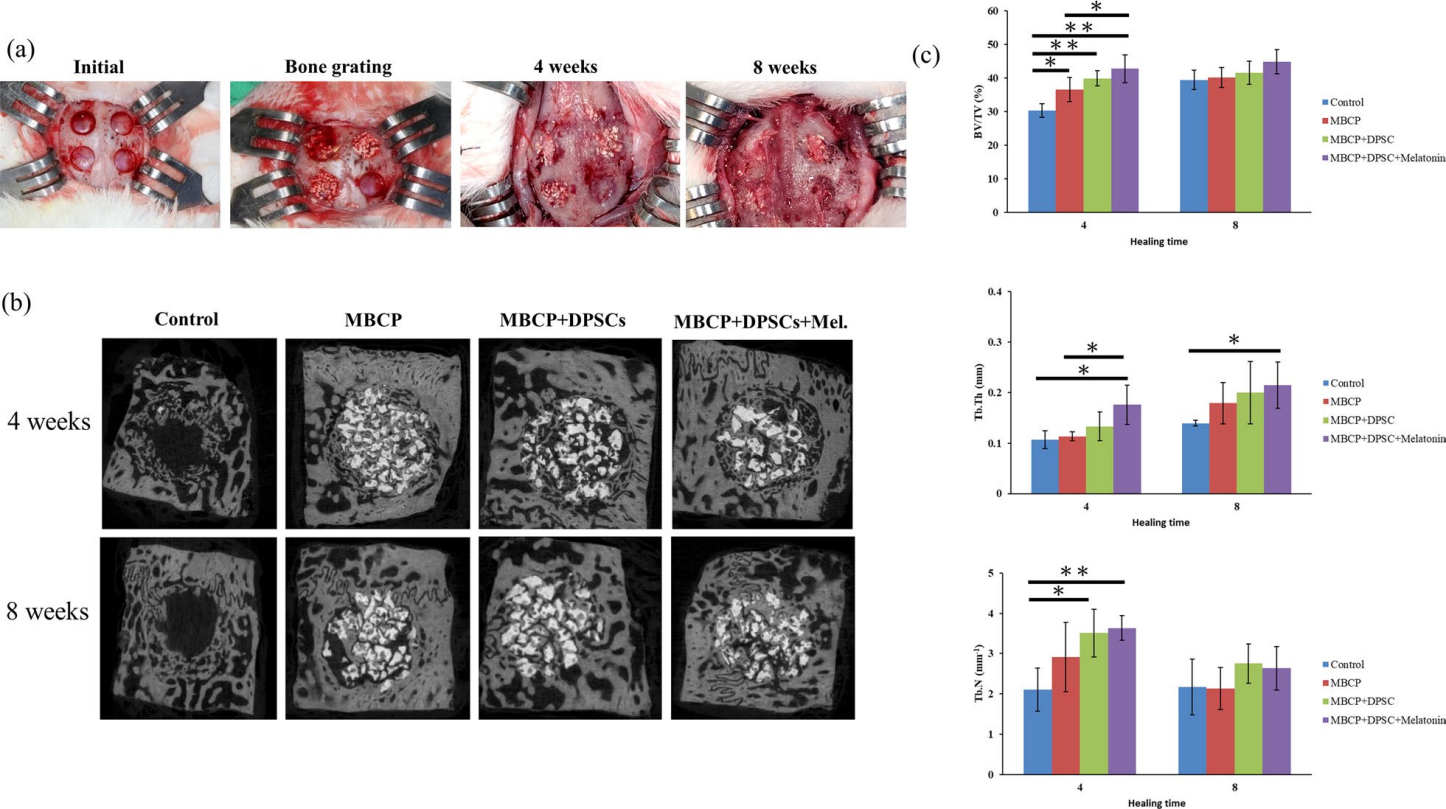
Clinical images of surgery, micro-CT scan, and three-dimensional reconstruction of the bone defects 4 and 8 weeks after implantation of the scaffold and DPSCs and statistical analysis. **a** Clinical images of surgery procedures and well healing status was presented at 4 and 8 weeks. **b** Sagittal planes of 3D reconstruction images of surgical bone defects after 4 and 8 weeks of scaffold and DPSCs implantation. **c** Bone histomorphometry based on micro-CT images, including bone volume (BV/TV), trabecular thickness (Tb.Th), and trabecular number (Tb.N). Results are expressed as mean ± standard deviation (SD) and statistical significance is represented as (*) *p* < 0.05 or (**) *p* < 0.01

Bone volume fraction and trabecular bone structural modeling were quantified using micro-CT analysis at 4 and 8 weeks after surgery. The clinical procedures of surgery are presented in Fig. 9a, and sagittal planes of 3D reconstruction images of surgical bone defects are illustrated in Fig. 9b. In the empty control group, only a small amount of new bone was observed at the border of the bone defect at 4 and 8 weeks. Newer bone formation was represented within the MBCP scaffold, while more bone bridge formation was demonstrated in both MBCP + DPSCs and MBCP + DPSCs + melatonin groups. The BV/TV values were significantly higher in the MBCP + DPSCs + melatonin group (42.75% ± 4.1%) than in the control (30.31% ± 1.9%), MBCP (36.5% ± 3.5%), and MBCP + DPSCs (39.9% ± 2.3%) groups (*p* < 0.05) at 4 weeks after surgery (Fig. 9c), suggesting the beneficial effects of melatonin preconditioning for DPSCs in promoting bone regeneration. However, no significant differences were observed among the experimental groups. Moreover, trabecular thickness (Tb.Th mm) and trabecular number (Tb.N mm^−1^) were significantly higher after implantation with melatonin-preconditioning DPSCs than in the empty control and MBCP scaffold-only groups (*p* < 0.05) (Fig. 9c).

**Fig. 10:**
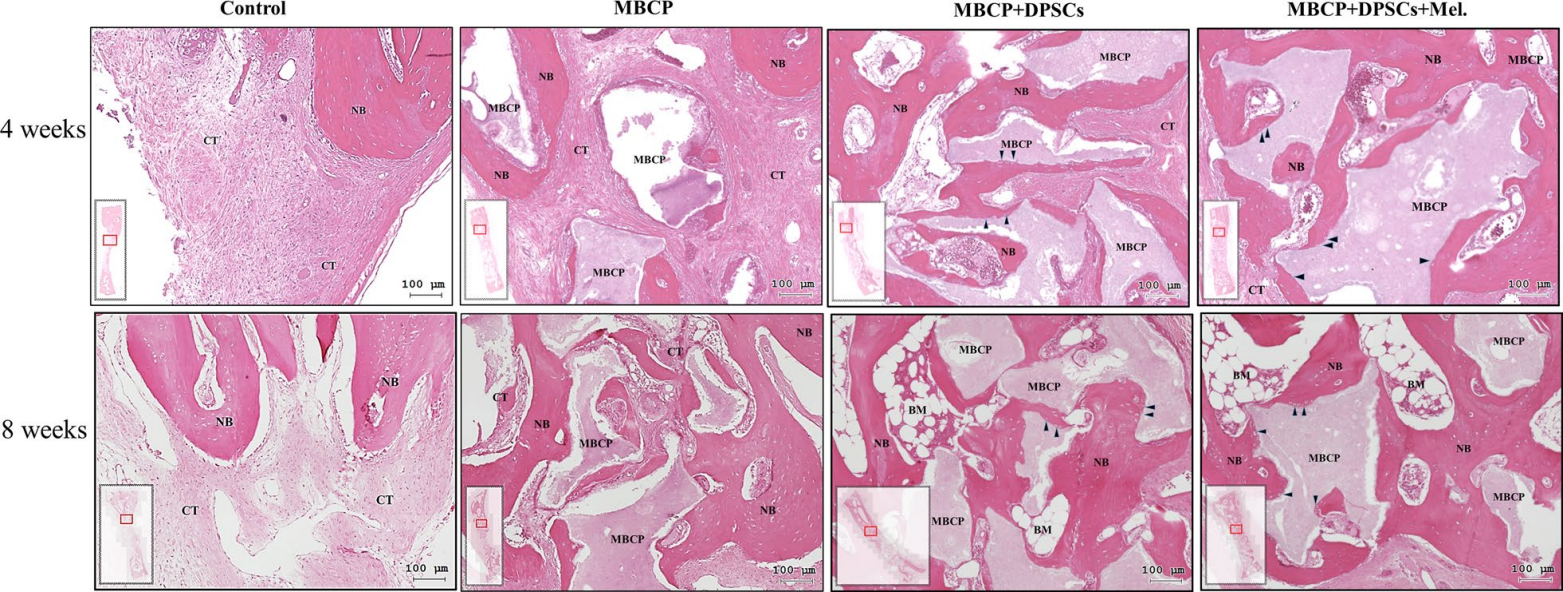
Melatonin-preconditioning DPSCs promoted bone defect healing in vivo. Histological analysis of regenerative tissues with H&E staining. The black arrowheads indicate osteoblasts at the MBCP–new bone interface. Scale bar = 100 μm. (*NB* new bone, *CT* connective tissue, *BM* bone marrow, *MBCP* scaffold, *DPSCs* dental pulp mesenchymal stem cells, *Mel* melatonin, *H&E* hematoxylin and eosin)

**Fig. 11:**
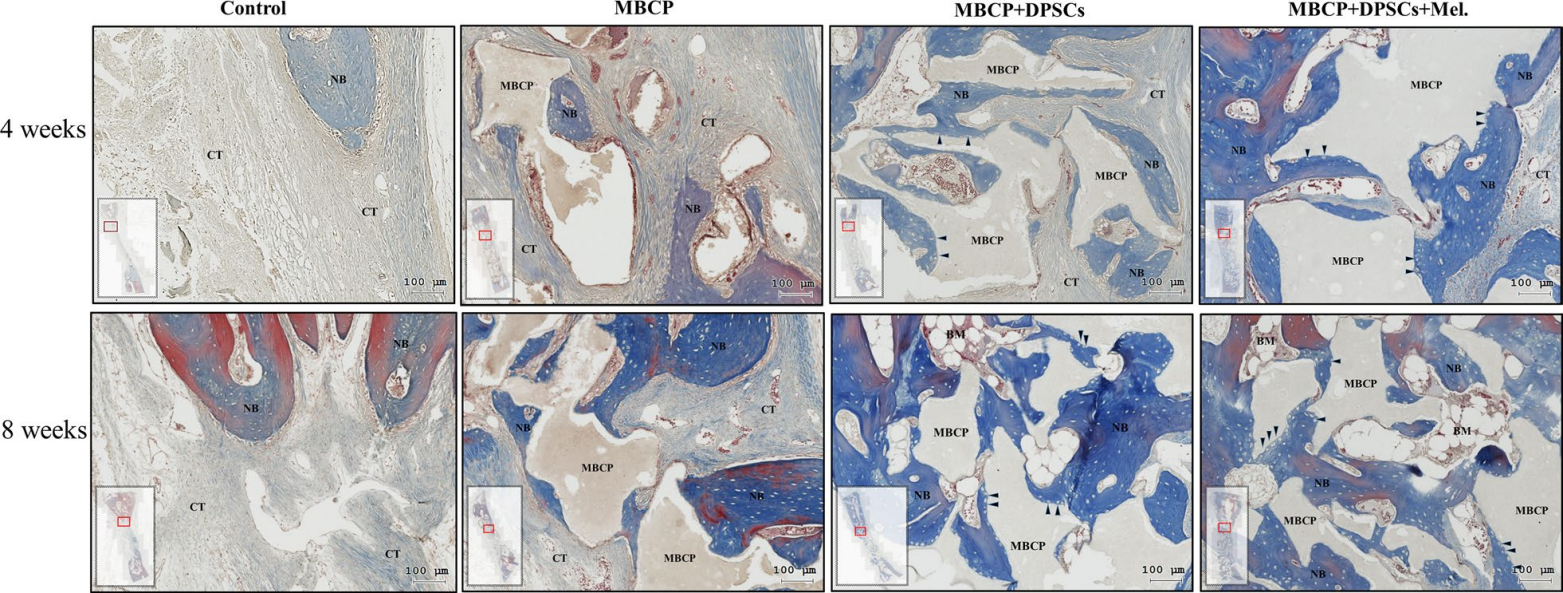
Melatonin-preconditioning DPSCs promoted bone defect healing in vivo. Histological analysis of regenerative tissues with Masson’s trichrome staining. The black arrowheads indicate osteoblasts at the MBCP–new bone interface. Scale bar = 100 μm. (*NB* new bone, *CT* connective tissue, *BM* bone marrow, *MBCP* scaffold, *DPSCs* dental pulp mesenchymal stem cells, *Mel* melatonin)

After micro-CT scanning, the calvarial bone specimens were sent for histological evaluation using hematoxylin–eosin (H&E) (Fig. 10), Masson’s trichrome (Fig. 11), immunohistochemical OCN (Fig. 13), and RUNX2 (Fig. 14) staining. As illustrated in Figs. 10 and 11, H&E staining and Masson’s trichrome staining of the bone defect sections displayed mainly fibrous connective soft tissue and limited new bone formation in the empty control group after 4 weeks of healing. In the MBCP group, little new bone was formed around the scaffolds. Upon treatment with DPSCs and melatonin-preconditioning DPSCs, more new bone formation and osteoblast lining on the surface of the newly formed bone were observed in the bone defect area. Moreover, more newly formed bone ingrowth into the scaffolds was presented in the MBCP + DPSCs + melatonin group. After 8 weeks of healing, some new bone formation was also observed in the empty control group, while a moderate amount of new bone regeneration was noted in the MBCP group. MBCP + DPSCs and MBCP + DPSCs + melatonin groups exhibited extensive solid bone structure, mature trabecular bone tissues, more bone bridges, and obvious bone marrow compared with the control and MBCP groups after 8 weeks of healing (Figs. 10, 11). Furthermore, the grafting materials were gradually resorbed and replaced by new bone, and more osteoblastic cells were lining on the surface of the newly formed bone and bone-grafting materials. These findings demonstrated that melatonin-preconditioning DPSCs can efficiently enhance the osteoconductive capacities of MBCP and bone remodeling turnover.

**Fig. 12:**
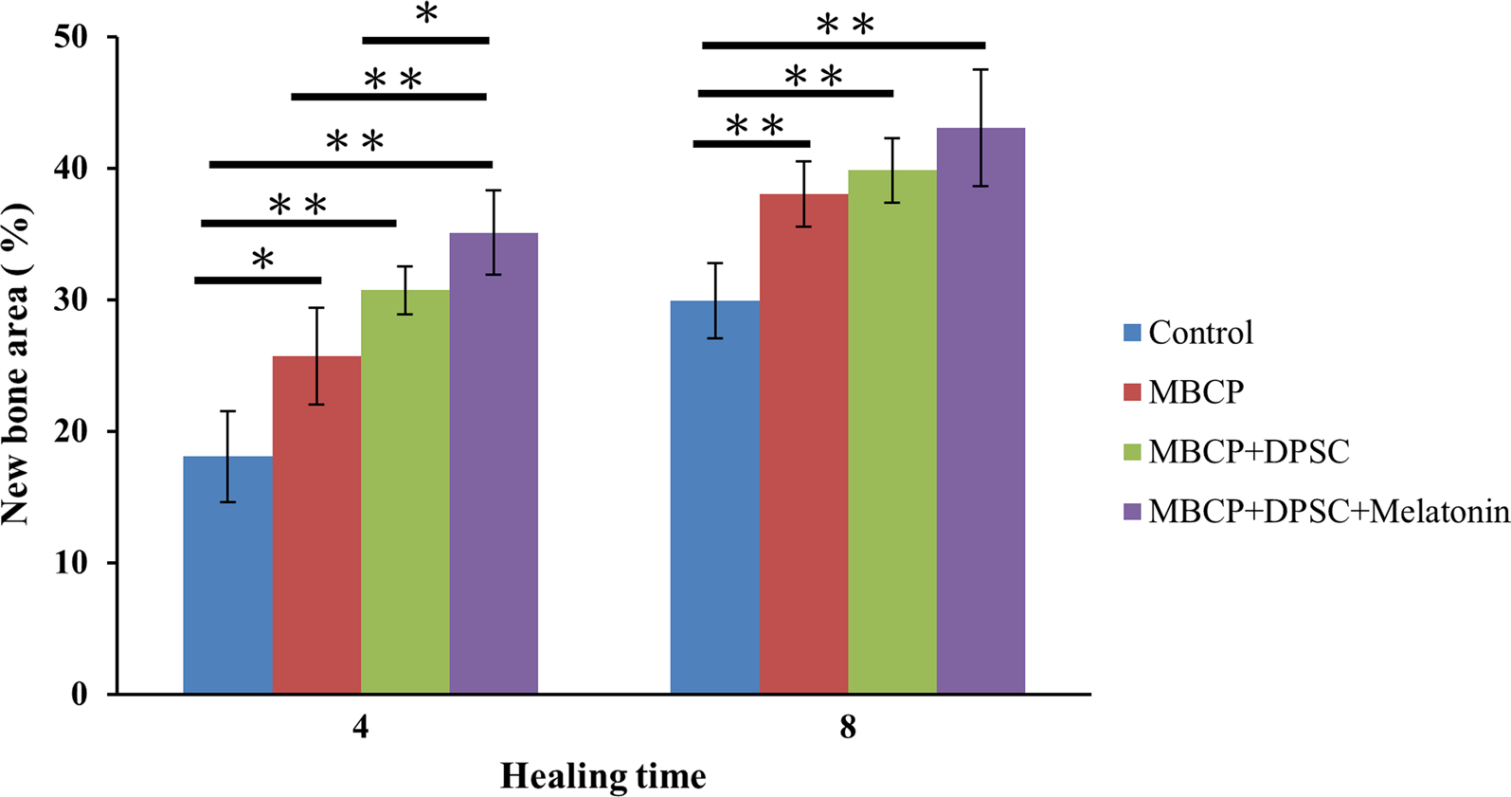
Histomorphometric analysis of new bone formation in bone defect area among empty control, MBCP only, MBCP + DPSCs, and MBCP + DPSCs + melatonin groups after 4 and 8 weeks of healing. Results are expressed as mean ± standard deviation (SD) and statistical significance is represented as (*) *p* < 0.05 or (**) *p* < 0.01

Quantitative histomorphometric analysis revealed that the MBCP + DPSCs (30.7 ± 1.8%) and MBCP + DPSCs + melatonin (35.1 ± 3.2%) groups demonstrated statistically higher percentages of new bone formation than the empty control (18.1 ± 3.4%) (*p* < 0.01) and MBCP only (25.6 ± 3.6%) groups (*p* < 0.05) after 4 weeks of healing (Fig. 12). A similar healing tendency was also presented at 8 weeks. MBCP only (38.0 ± 2.4%), MBCP + DPSCs (39.8 ± 2.4%), and MBCP + DPSCs + melatonin (43.0 ± 4.4%) groups demonstrated a statistically higher percentage of new bone formation than empty control (29.9 ± 2.8%) (*p* < 0.01). However, no significant difference was found among the MBCP only, MBCP + DPSCs, and MBCP + DPSCs + melatonin groups.

**Fig. 13:**
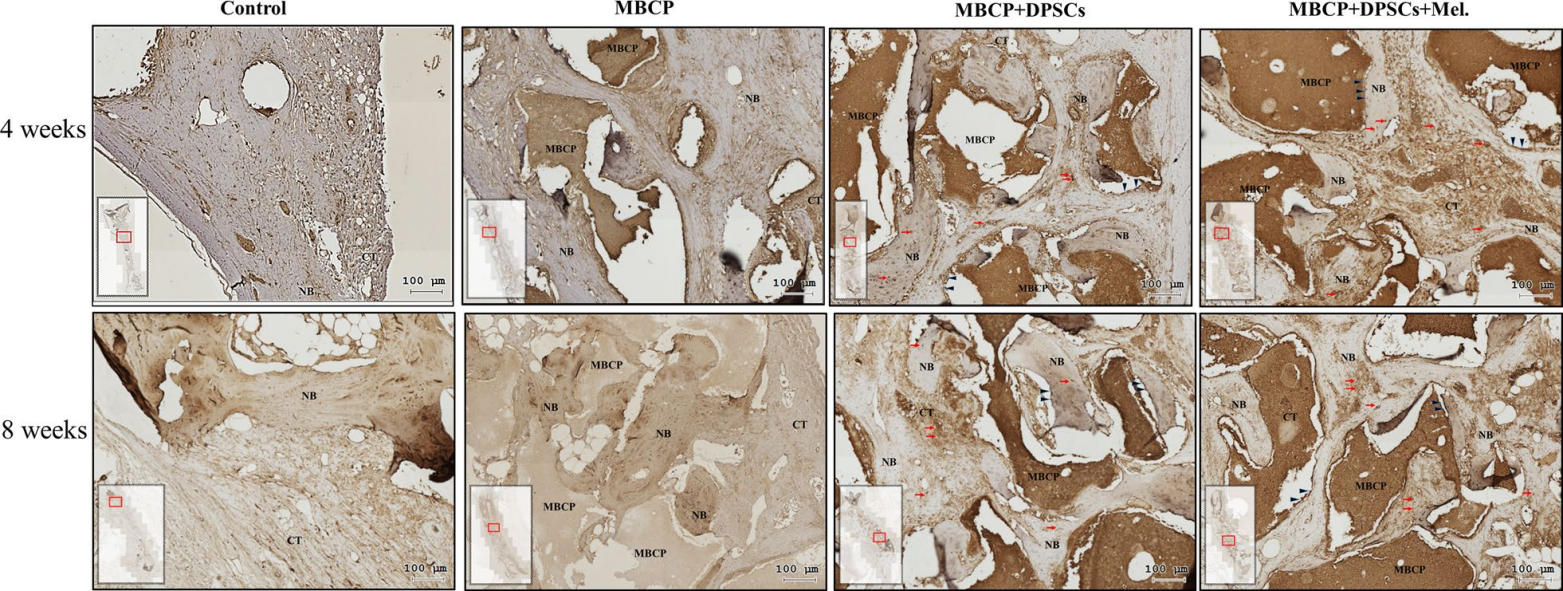
Immunohistochemistry staining was employed to detect the expression of OCN in the bone defect area. The black arrowheads indicate OCN-positive osteoblasts at the MBCP–new bone interface. The red arrows indicate a high expression region and OCN-positive osteocytes embedded within the bone and connective tissue matrix. Scale bar = 100 μm. (*NB* new bone, *CT* connective tissue, *BM* bone marrow, *MBCP* scaffold, *DPSCs* dental pulp mesenchymal stem cells, *Mel* melatonin, *OCN* osteocalcin)

**Fig. 14:**
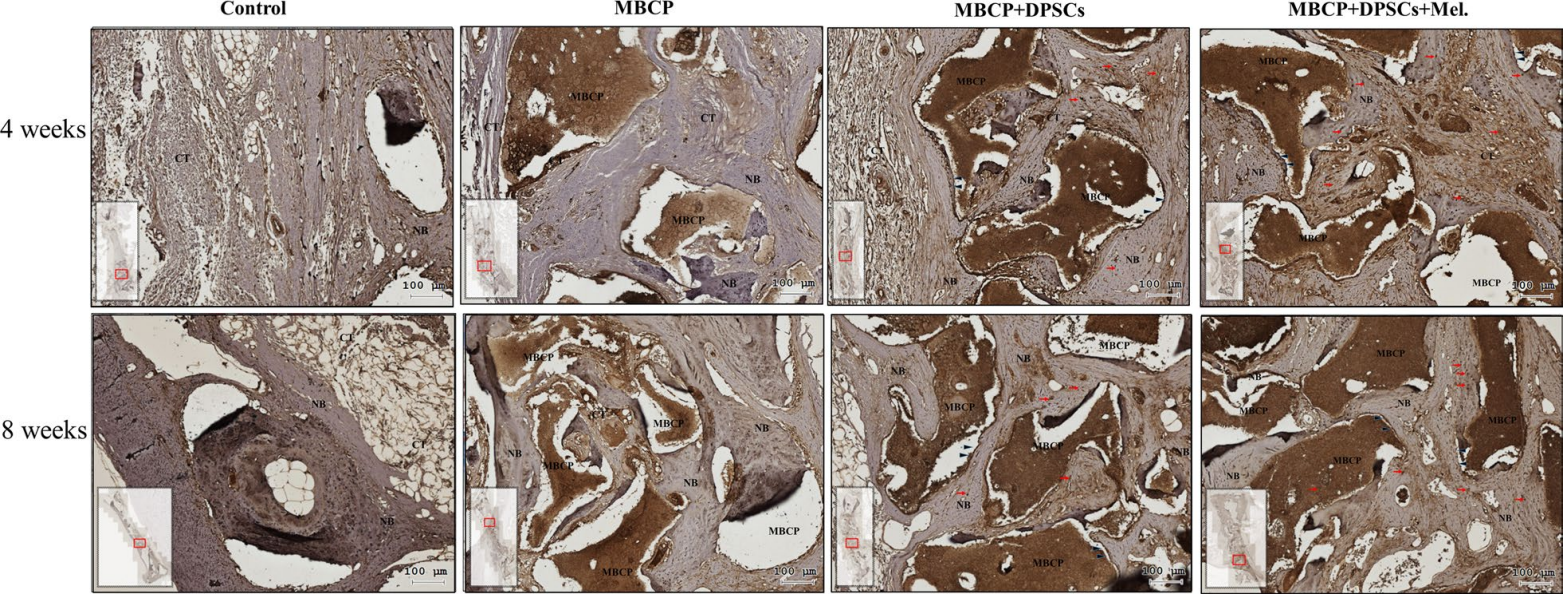
Immunohistochemistry staining was employed to detect the expression of RUNX2 in the bone defect area. The black arrowheads indicate RUNX2-positive osteoblasts at the MBCP–new bone interface. The red arrows indicate a high expression region and RUNX2-positive osteocytes embedded within the bone and connective tissue matrix. Scale bar = 100 μm. (*NB* new bone, *CT* connective tissue, *BM* bone marrow, *MBCP* scaffold, *DPSCs* dental pulp mesenchymal stem cells, *Mel* melatonin)

Similarly, immunohistochemical staining demonstrated that both DPSCs and melatonin-preconditioning DPSCs can efficiently promote the expression of the OCN and RUNX2 protein markers. OCN and RUNX2 were weakly expressed in the empty group and MBCP-only groups. As illustrated in Figs. 13 and 14, abundant OCN and RUNX2-positive osteoblasts were presented at the MBCP–new bone interface, and OCN and RUNX2-positive osteocytes were extensively embedded within the bone and connective tissue matrix after the treatments of DPSCs and melatonin-preconditioning DPSCs. The OCN and RUNX2 immunostaining results suggested that the local application of melatonin-preconditioning DPSCs can synergistically promote osteogenesis, bone regeneration, osteoinduction, and bone remodeling in the bone defect area.

## Discussion

Although MSC-based therapy has shown promising outcomes of tissue regeneration in preclinical models and clinical trials in the last few decades, the efficiency of MSC transplantation and survival of engrafted stem cells remain a major challenge, especially in disease, aging, and inflammatory conditions. Recently, preconditioning strategies have been proposed for the enhancement of MSC-based therapy, including growth factors, gene regulation, mechanical stimulation, and small molecules. Small molecules have the advantages of safety, effectiveness, rapid response, easy acquisition, reversibility, low harm, low cost, and high efficiency. Moreover, the effect of small molecules is concentration dependent [19, 21]. The more commonly used small molecules include statins, oxysterols, resveratrol, bisphosphonates, metformin, and melatonin [44]. Among them, melatonin has gradually attracted attention for enhancing cell activities and preserving the functional integrity of MSCs via the regulation of reactive oxygen species generation and anti-inflammatory reaction [25, 45, 46]. Although melatonin can promote osteogenic differentiation of osteoblasts and BMSCs, a comprehensive analysis of the effect of melatonin on DPSCs and the signaling pathways involved remains unknown. Therefore, we evaluated the effects of melatonin on proliferation, surface marker expression, osteogenic differentiation, mineralization, and underlying molecular signaling pathways of DPSCs in vitro and assessed new bone formation capacities of melatonin-preconditioning DPSCs in calvarial bone defects.

Our results revealed that 100 and 10 μM melatonin exerted positive effects on the proliferation without influencing the cell morphology after 3 days of culture (Fig. 2). These results were inconsistent with studies reporting that 100 μM melatonin improves synovial MSC proliferation under inflammation conditions and enhances DPSC migration and proliferation [47, 48]. However, melatonin has no significant or inhibitory effects on dental papilla cell proliferation [39, 49]. Thus, melatonin’s positive effects on MSC proliferation depend on MSC type and melatonin concentration [25, 37].

In the present study, DPSCs with and without melatonin treatment were all positive for MSC markers (CD44, CD90, CD105, and CD146) and negative for hematopoietic stem cell markers (CD34 and CD45). These results were inconsistent with previous DPSC studies [5, 8, 11]. Notably, DPSCs treated with 100 μM melatonin presented a significantly higher expression of CD146 surface markers (Fig. 3). One study demonstrated that CD146-positive DPSCs can promote self-renewal and mineralization and generate dentin/pulp-like structures in immunocompromised mice [50]. CD146 + MSCs from umbilical cords have greater potency for cartilage protection by suppressing Th17 cell activation, and CD146 + perivascular BMSCs would more robustly have higher immunomodulatory, anti-inflammatory, and secretory capacities [51, 52]. Thus, melatonin can efficiently promote the DPSC expression of CD146, which is associated with increasing self-replication and immunomodulation [50].

To identify the effects of melatonin on osteogenic differentiation and mineralization of DPSCs, ALP activity, Alizarin Red S staining, and expression of osteogenic genes were evaluated. ALP activity, an early osteogenic differentiation marker, can be enhanced when DPSCs are exposed to melatonin in a concentration-dependent manner. In particular, the ALP activity of DPSCs without osteogenic induction can also be promoted by higher concentrations of melatonin. These results were consistent with one study in which 100 μM melatonin upregulated the ALP activity of MC3T3-E1 cells under hypoxic conditions [45]. In the present study, melatonin promoted mineralization of DPSCs with osteogenic differentiation in time- and concentration-dependent manners (Fig. 5). The expression of osteogenic genes in DPSCs was also enhanced after 3-day exposure to melatonin (Fig. 6). Overall, our results indicated that 100 μM melatonin can efficiently promote osteogenic differentiation and mineralization of DPSCs. Different concentrations of melatonin—from physiological concentrations (50 nM) to higher concentrations (200 μM)—have been observed to positively promote osteogenic differentiation of osteoblasts, MSCs, BMSCs, DPSCs, and dental papilla cells [34, 39, 45, 47, 53, 54]. Moreover, melatonin can synergistically enhance the BMP family-induced osteogenic differentiation of osteoblasts [34, 53].

In the present study, RUNX2 gene expression of DPSCs with and without osteogenic induction was significantly upregulated by 100 μM melatonin. Among those transcriptional factors related to osteogenesis and MSC differentiation, Runx2 is crucial for regulating MSCs to the osteoblast lineage and regulating the expression of osteoblast-specific genes (collagen I, bone sialoprotein, osteopontin, and osteocalcin) in the early stages of differentiation [55–58]. Runx2 deficiency in mice resulted in severe bone defects in the calvaria and long bone, suggesting that Runx2 is essential in both intramembranous and endochondral ossification [59]. Furthermore, stabilizing Runx2 gene expression and maintaining Runx2 protein levels can mediate and increase physiologic bone formation [60]. Thus, Runx2 may be the best therapeutic model gene for identifying melatonin-mediated osteogenesis and osteogenic differentiation of DPSCs.

Although many signaling pathways are involved in the melatonin-mediated osteogenic differentiation of osteoblasts or BMSCs, the molecular mechanisms involved in the melatonin-mediated osteogenic differentiations of DPSCs remain unclear [61]. Melatonin enhances osteoblastic differentiation of MC3T3-E1 cells via the BMP/ERK/Wnt signaling pathways and by activating PKD/p38 signaling pathways under hypoxic conditions [33, 45]. Moreover, melatonin could promote osteoblastic differentiation in MC3T3-E1 cells by activating the PDGF/AKT signaling pathway and enhancing fracture healing in a mouse femoral fracture model [61]. Our study is the first to demonstrate that melatonin can dose-dependently regulate NF-κB/COX-2 and MAPK signaling pathways of DPSCs. The ALP, OCN, and Runx2 protein levels confirmed the osteogenesis-promotion effect of melatonin on DPSCs, especially at the 100 μM dosage. Notably, the increase in the COX-2 and NF-κB protein expression of DPSCs during osteogenic differentiation was attenuated by 100 μM melatonin. This is also consistent with Zhou et al. (2020) [32], who reported that melatonin significantly accelerates the osteogenic differentiation of BMSCs by suppressing the NF-κB signaling pathway and inhibits osteoclastogenesis by suppressing RANKL production. This is probably because the inhibition of COX-2 and NF-κB activation are reflected by downregulation of proinflammatory and upregulation of anti-inflammatory cytokines, which is crucial to tissue homeostasis and regeneration [62].

Moreover, the protein expression of p-p38 and p-ERK in DPSCs with osteogenic induction was also significantly activated by 100 μM melatonin, while melatonin had no significant effect on the p-JNK expression of DPSCs (Fig. 7c, d). Furthermore, the increase in mineralization and osteogenesis-related gene expression of DPSCs by melatonin was attenuated by the inhibitors of p38 (SB203580) and ERK (PD98059), as presented in Fig. 8. Thus, p-p38 and p-ERK are important mediators in the melatonin-mediated osteogenic differentiation of DPSCs. This is because MAPKs play a critical role in regulating MSC proliferation, differentiation, and apoptosis and are involved in multiple cellular signaling pathways [57, 63, 64].

Among the three classic MAPKs, p38 and ERK play crucial roles in osteogenic differentiation of MSCs, bone development/homeostasis, and bone repair/regeneration [31, 57, 63]. JNK signaling is essential for self-renewal and stemness maintenance of the undifferentiated status of pluripotent and embryonic stem cells [65]. Our results demonstrated that the regulation of osteogenic differentiation by melatonin in DPSCs is mediated through cross talks with COX-2/NF-κB and p38/ERK signaling pathways, but not through JNK signaling.

In the in vivo study, obvious new bone formation and osteogenic protein expression was observed in the MBCP + DPSCs + melatonin group. Compared with the control group, MBCP, and MBCP + DPSCs, the MBCP + DPSCs + melatonin group presented significantly new bone regeneration (BV/TV) and trabecular bone structural modeling (Tb.Th and Tb.N mm) on micro-CT analysis (Fig. 9). Moreover, significant quantities of new bone formation and osteogenic protein expression were demonstrated in the MBCP + DPSCs + melatonin group, which presented more new bone formation and osteoblast lining at the early healing stage and more solid bone structure, mature trabecular bone tissues, more bone bridges, and obvious bone marrow at the remodeling stage (Figs. 10, 11). In addition, OCN and RUNX2 immunostaining revealed that melatonin-preconditioning DPSCs combined with MBCP synergistically and efficiently accelerated osteoinduction and bone remodeling in the bone defect area (Figs. 13, 14). One data also revealed that preconditioning with melatonin improves the ectopic osteogenesis and calvarial defect repair of long-term passaged BMSCs by activating the antioxidant defense system and preserving the stemness capacities [17]. This is because preconditioning with melatonin not only maintains the self-renewal characteristics and promotes osteogenic differentiation of DPSCs but also plays a powerful redox regulator and a cellular protective role in preventing cellular damage from local inflammation, oxidative stress, and hypoxic environments [25, 30, 66]. However, the activated DPSCs contribute to bone healing via direct cell-to-cell interaction/communication or by indirect factor secretion, including cytokines, growth factors, extracellular vesicles, and exosomes within the defect area, which trigger the regulatory signals and provide a complex microenvironment or niche for mediating tissue regeneration [9, 67]. These results demonstrated that melatonin-preconditioning DPSCs loaded in MBCP bone-grafting materials can efficiently enhance bone regeneration and bone remodeling turnover in calvarial defects. To our knowledge, this is the first study to present a melatonin-preconditioning strategy to improve DPSCs therapy in osteogenesis, bone regeneration, and bone remodeling in vivo. Our proposed method is efficient and should be further evaluated for potential clinical application.

## Conclusion

Our results demonstrated that melatonin can efficiently promote the osteogenic differentiation of DPSCs by regulating COX-2/NF-κB and p38/ERK MAPK signaling pathways. Furthermore, DPSCs preconditioning with 100 μM melatonin can successfully accelerate bone healing and bone remodeling turnover in calvarial bone defects. Thus, preconditioning with melatonin on can serve as an efficient strategy for improving MSCs osteogenesis and bone regeneration in vivo.

## Data Availability

The data during the current study are available from the corresponding author on a reasonable request.

## Abbreviations

MSC: Mesenchymal stem cell
DPSCs: Dental pulp-derived MSCs
ALP: Alkaline phosphatase
BMSCs: Bone marrow-derived MSCs
PBS: Phosphate-buffered saline
GM: Growth medium
MAPK: Mitogen-activated protein kinase
DMSO: Dimethylsulfoxide
OM: Osteogenic differentiation medium
FITC: Fluorescein isothiocyanate
pNPP: *p*-Nitrophenyl phosphate
BMP-2: Bone morphogenetic protein-2
OCN: Osteocalcin
RUNX2: Runt-related transcription factor 2
GAPDH: Glyceraldehyde 3-phosphate dehydrogenase
RIPA: Radioimmunoprecipitation assay
MBCP: Micro–macro biphasic calcium phosphate
CT: Micro-computed tomography
3D: Three-dimensional
BV/TV%: Bone volume density (bone volume/total tissue volume)
Tb.Th: Trabecular thickness
Tb.N: Trabecular number
HE: Hematoxylin–eosin
